# Midodrine initiation criteria, dose titration, and adverse effects when administered to treat shock: A systematic review and semi-quantitative analysis

**DOI:** 10.2478/jccm-2025-0007

**Published:** 2025-01-31

**Authors:** Madeleine M. Puissant, Kaitlin J Armstrong, Richard R Riker, Samir Haydar, Tania D Strout, Kathryn E Smith, David B Seder, David J Gagnon

**Affiliations:** Maine Medical Center, Portland, USA

**Keywords:** midodrine, shock, sepsis, critical care, systematic review

## Abstract

**Objective:**

Systematically examine the literature describing midodrine to treat shock and to summarize current administration and dosing strategies.

**Data sources:**

Structured literature search conducted in MEDLINE (PubMed) from inception through May 10, 2023.

**Study Selection and Data Extraction:**

Abstracts and full texts were assessed for inclusion by two blinded, independent reviewers. English-language publications describing use of midodrine in adult patients with shock were included. Data were extracted by two blinded, independent abstractors using a standardized extraction tool. Quality assessments were completed by paired reviewers using JBI methodology.

**Data Synthesis:**

Fifteen of 698 (2%) screened manuscripts were included with 1,714 patients with a variety of shock types. Seven studies (47%) were retrospective, two (13%) prospective observational, and six (40%) randomized controlled studies. Midodrine was initiated to facilitate intravenous vasopressor (IVP) weaning in most (11, 73%) studies; only two (13%) reported IVP weaning protocol use. Starting doses were 10 mg every 8 hours (4, 27%) or three times a day (3, 20%), 20 mg every 8 hours (2, 13%); six studies (40%) did not report initial midodrine dosing. A midodrine titration protocol was reported in 6 (40%) studies. Thirteen (87%) studies evaluated for bradycardia, identified in 6 (46%) studies among 204 patients; only one (0.5%) patient required midodrine discontinuation. Three (20%) studies reported on hypertension with an incidence of 7–11%. Four (27%) studies assessed for ischemia; 5/1128 (0.4%) patients experienced mesenteric ischemia requiring midodrine discontinuation.

**Relevance to Patient care and Clinical Practice:**

This review explores the pragmatic details involved in initiating, titrating, and weaning midodrine for the bedside clinician and identifies rates of adverse events and complications.

**Conclusions:**

Published literature describing midodrine use for shock is heterogeneous and comprised primarily of low or very low quality data. Future controlled trials addressing the shortcomings identified in this systematic review are warranted.

## Introduction

Midodrine is an oral alpha-1 receptor antagonist that was approved for the treatment of symptomatic ortho-static hypotension by the Food and Drug Administration (FDA) in 1996 [1]. Since then, it has been utilized off-label for blood pressure augmentation in multiple diagnoses, including shock, and its use in the critically ill has increased seven-fold in the past decade [2]. Despite expanding use, important pragmatic issues such as initiation threshold, dose titration parameters, and the clinical relevance of adverse drug effects remain poorly defined.

Midodrine's prescribing information for orthostatic hypotension recommends a starting dose of 10 mg by mouth three times daily during waking hours to avoid persistent systolic supine hypertension [1]. Single doses of 20 mg and daily doses greater than 30 mg may be tolerated [1]. In the setting of shock, single doses as high as 40 mg, and total daily doses of 120 mg, have been reported in the literature most commonly with every 8 hour dosing intervals [3,4]. Though approved for titration to desired blood pressure with confirmed dose-response effects, many studies have used a fixed-dose regimen which may limit midodrine's effectiveness [1,5,6,7]. The discrepancies between the prescribing information and recent clinical practice warrants further study.

We are aware of three published meta-analyses that aggregated midodrine effectiveness data from only randomized-controlled trials [8,9,10]. Although randomized-controlled trials (RCTs) are generally considered high quality data, many publications included in prior meta-analyses used fixed-dose approaches without intravenous vasopressor (IVP) weaning protocols and variable outcome criteria. The meta-analyses also focused on clinical outcomes and safety, leaving pragmatic questions unanswered including initiation thresholds, dose titration strategies, and the clinical relevance of adverse drug effects. If these questions can be resolved, the potential for midodrine to decrease ICU length of stay, cost of care, and complications of IVPs may be realized.

The objective of this systematic review and semi-quantitative analysis was to assess a broader array of published studies to document administration and dosing practices with the goal of improving bedside practice and informing the potential design of future controlled trials.

## Methods

### Publication Identification

A structured search of MEDLINE (PubMed) identified all English-language publications with “midodrine” in the title or abstract from inception through May 10, 2023. Publications that met predefined patient, intervention, comparator, and outcome (PICO) criteria were screened for full-text review: *Patients* (adults ≥18 years of age with shock); *Intervention* (midodrine); *Comparator* (not required; studies with and without control groups were included); and *Outcomes* (midodrine dosing and adverse drug effects).

Two investigators (TDS and DJG) independently screened titles and abstracts for evaluation with a third investigator (RRR) available for disagreements. Publications were excluded if they treated a diagnosis other than shock (e.g., orthostatic hypotension) or were a case report, trial protocol, letter to the editor, conference abstract, systematic review or meta-analysis. Publication references were evaluated during the full-text review.

### Data Extraction

**Fig. 1.**
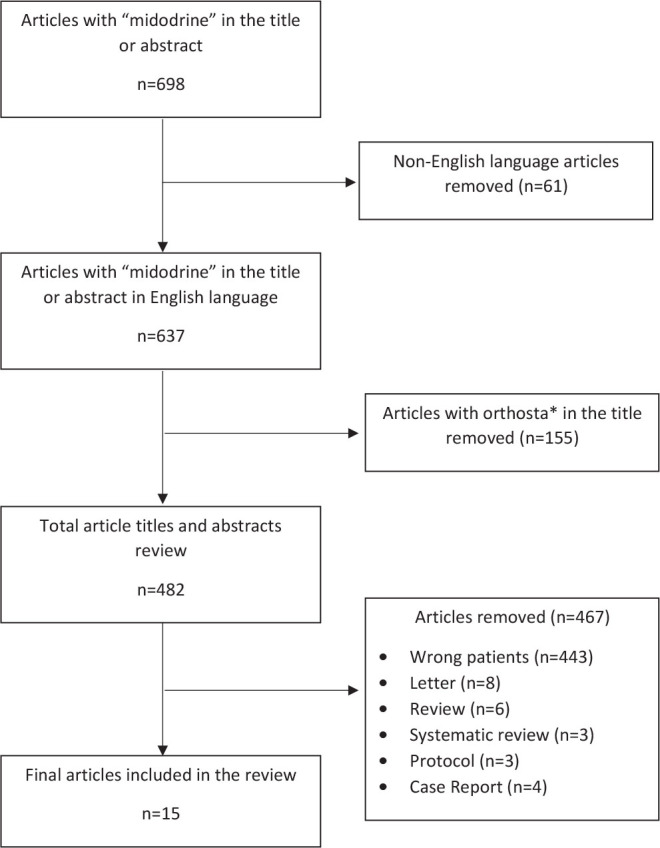
Preferred reporting items for systematic reviews and meta-analyses (PRISMA) flow diagram

Data were extracted by two investigators (MMP and KJA) using a standardized template (Figure 1). No protocol was published for this systematic review, but consensus guidance was followed including the Preferred Reporting Items for Systematic review and Meta-Analysis Protocols (PRISMA-P).

### Patient Characteristics

Demographic and clinical characteristics included study design, country, patient population including rurality, severity of illness (e.g., APACHE II), shock etiology, renal function at the time of midodrine initiation and during therapy, phase of care (i.e., emergency department or ICU), ICU and hospital length of stay, and mortality.

### Midodrine Administration

Midodrine administration data included use of a midodrine dosing protocol, initial and maximum dose and frequency, dosing strategy (titrated or fixed), renal dose adjustments, timing of initiation (before, with, or after IV vasopressors), duration of therapy, route of administration (oral or feeding tube) and continuation at ICU and hospital discharge.

### Intravenous Vasopressors

Vasopressor data included dosage and frequency of administration, weaning protocols, number of patients on IVPs at the time of midodrine initiation, central venous catheter duration and complications (e.g., central line-associated bloodstream infections), complications related to IVPs (e.g., extravasation), and time to IVP discontinuation. Vasopressor doses were converted to norepinephrine equivalents as previously described [11].

### Adverse Drug Effects

Potential adverse drug effects were determined a priori including bradycardia, bowel or limb ischemia, and stroke. Definitions were according to the study under review and are referred to in this manuscript as present or absent, accordingly.

### Cost Analyses

Cost analysis data included direct medical costs per day of patients administered midodrine versus those receiving standard care.

### Missing Data

If a data point was not evaluated in a publication, it was classified as “not reported,” and if it was evaluated for but not observed, it was classified as “not observed.” Corresponding authors for publications with missing data were contacted by e-mail, when appropriate.

### Quality of Evidence Assessment

Study quality was assessed by two blinded reviewers (MMP and TDS) using the JBI Critical Appraisal Checklists for RCTs, case control studies, case series, and cohort studies [12,13,14]. Studies were evaluated for their methodologic rigor and for potential bias in their design, conduct, and analysis. Initial, pre-discussion interrater agreement on quality appraisal was 0.84, 95% CI: 0.758–0.918 using Cohen's kappa statistic. A consensus process was then used to come to a final decision on initial disagreements.

### Statistical Analysis

Continuous data are reported as median (interquar-tile range 25^th^ – 75^th^ percentile), and categorical or dichotomous data as number and percentage. This study reports semi-quantitative data; quantitative analyses were not performed given the study objectives and the heterogeneity of aggregated data.

## Results

### Study Characteristics

A total of 698 publications were identified and 15 (2%) were included (Figure 1) [2,3,4, 15,16,17,18,19,20,21,22,23,24,25,26]. Midodrine was administered to 1,714 patients with a median of 31 (20–79) patients per study. The first study included patients treated as early as February 2012, with the most recent study including patients treated through April 2021 [15,26]. Seven (47%) studies were retrospective, two (13%) were prospective observational, and six (40%) were RCTs; four of the six (67%) RCTs were open-label. Most studies (12/15; 80%) were single center and conducted in the United States (9/15; 60%) (Table 1). The primary outcome was time to IVP discontinuation in nine (60%) studies.

**Table 1.** Design of included studies

**Study**	**Design**	**Country**	**Inclusion Criteria**	**Exclusion Criteria**	**Primary Outcome**
Ahmed Ali 2022	RCT; blinding unclear; single center	Egypt	Spinal shock in the ICU; age ≥18 years; hemodynamically stable on low-dose NE (<8 mcg/min) monotherapy	Anuric or oliguric; CKD; allergy	Total duration of IVP
Costa-Pinto 2022	Pilot RCT; open-label; multicenter	Australia and New Zealand	Admitted to the ICU; age ≥18 years; clinically stable with hypotension for >24 hours requiring low-dose IVP (≤10 mcg/min of NE or ≤100 mcg/min of metaraminol) monotherapy	Lactate >4 mmol/L; renal failure; hemorrhagic, obstructive, or cardiogenic shock; liver failure; severe heart disease; acute brain pathology; pregnancy; thyrotoxicosis; bradycardia (HR <50 bpm), NPO or fed via jejunal tube; allergy	Time from randomization to discontinuation of IVP
Davoudi-Monfared 2021	Pilot RCT; open-label, single-center	Iran	Septic shock (MAP <65 mmHg and lactate ≥2 mg/dL despite fluid resuscitation) in the ICU; age ≥18 years; requiring IVP	≥24 hours since septic shock onset; CKD (GFR <30 mL/min); neurogenic bladder and urination disorders; PAD; scleroderma, bradycardia (HR <60 bpm); MID PTA	Lactate clearance at 4, 24 and 48 hours
Hussein El Adly 2022	RCT; open label; single-center	Egypt	Septic shock in the ICU; age 18–80 years; hypotension (SBP <90 mmHg and MAP <65 mmHg) for >24 hours requiring IVP	Hypovolemic shock; HF (EF <30%); CKD (SCr >2 mg/dL); thyrotoxicosis; pheochromocytoma; CMO; DDI (MAOIs, alpha-1 blockers, TCAs); orthostatic hypotension; bradycardia (HR <50 bpm); MID PTA; NPO; allergy	Total duration of IVP; duration of IVP wean; cumulative dose of IVP
Kim 2021	Retrospective cohort study; single center	USA	Patients admitted to ICU from ED then transferred to floor	ICU mortality; admitted to ICU due to diabetic ketoacidosis or tissue plasminogen activator administration	ICU readmission; rapid response team activation; hospital LOS; in-hospital mortality; 30 day hospital readmission
Lal 2021	Pilot RCT; double-blinded; multicenter	USA; United Arab Emirates	Septic shock (MAP <70 mmHg and SBP <130 mmHg despite antibiotics and fluids 30 mL/kg) in the ICU; age ≥18 years	ACS or EF <30%; GIB; obstructive or cardiogenic shock; lactate > 4 mmol/L; acute intraabdominal process; transferred from outside facility; cardiac arrest; child-bearing age; thyrotoxicosis; pheochromocytoma; PAD or ischemic bowel; CMO; DDI (MAOIs); bradycardia (HR <40 bpm); MID PTA; NPO; allergy	Duration of IVP in the first 24 hours
Levine 2013	Prospective cohort study; single-center	USA	Admitted to the SICU; age ≥18 years; clinically stable (otherwise discharge ready) with hypotension for >24 hours requiring low-dose IVP (phenylephrine <150 mcg/min or NE <8 mcg/min)	Hypovolemic shock; adrenal insufficiency; <3 doses of MID; orthostatic hypotension; MID PTA	Time from MID initiation to discontinuation of IVP; Change in IVP rate before/after MID initiation
Macielak 2021	Retrospective cohort study; single center	USA	Age ≥18 years; receiving MID dosed “four times daily” or “every six hours”	Incarcerated; pregnancy	Characterization of patients receiving MID “four times daily” or “every six hours”
Poveromo 2016	Retrospective cohort study; single-center	USA	Admitted to the ICU with diagnosis related to cardiovascular, trauma, or sepsis; age ≥18 years; requiring ≥1 IVP	ICU mortality within 24 hours; duration of IVP <2 hours; <3 doses of MID; MID for indication other than IVP weaning	Time from MID initiation to discontinuation of IVP
Rizvi 2018	Retrospective case series; single-center	USA	Admitted to the ICU; age ≥18 years; initiated on MID	MID PTA	Cumulative dose of IVP at MID initiation and 24 hours; MAP at MID initiation and 24 hours
Rizvi 2019	Retrospective case series; single-center	USA	Admitted to the ICU; age ≥18 years; initiated on MID	ICU mortality; MID PTA	Incidence of MID continuation after ICU discharge
Santer 2020	RCT; double-blinded; multicenter	USA, Australia	Admitted to the ICU or step-down unit; age ≥18 years; clinically stable with hypotension for >24 hours requiring low-dose (<100 mcg/min phenylephrine, <8 mcg/min of NE, or <60 mcg/min of metaraminol) IVP monotherapy	Clinical evidence of inadequate tissue oxygenation; adrenal insufficiency; liver failure; CKD (SCr >2 mg/dL); HF (EF <30%); acute urinary retention; pheochromocytoma; thyrotoxicosis; pregnancy; bradycardia (HR <50 bpm); MID PTA; NPO; allergy	Time from randomization to discontinuation of IVP
Tremblay 2020	Retrospective propensity matched cohort study; single center	Canada	Admitted to the ICU following cardiac surgery requiring CPB; age ≥18 years; hypotension requiring IVP for >12 hours post-surgery	MID before surgery; mechanical circulatory support before surgery; emergency surgery; transplantation; cirrhosis	Number of days alive and free from ICU at 30 days
Whitson 2016	Retrospective cohort study; single-center	USA	Septic shock in the ICU; clinically stable with hypotension for >24 hours requiring IVP	NR	Total duration of IVP; ICU LOS
Wood 2022	Retrospective case-control; single center	Australia	Admitted to ICU or step-down unit; age ≥18; clinically stable with hypotension for >24 hours requiring low-dose (<8 mcg/min of NE or <60 mcg/min of metaraminol) IVP monotherapy	Clinical evidence of inadequate tissue oxygenation; adrenal insufficiency; liver failure; CKD (SCr >2 mg/dL); HF (EF <30%); acute urinary retention; pheochromocytoma; thyrotoxicosis; pregnancy; bradycardia (HR <50 bpm); NPO; allergy	Time from intervention to discontinuation of IVP

Abbreviations: bpm, beats per minute; CPB, cardiopulmonary bypass; CKD, chronic kidney disease; DDI, drug-drug interaction; EF, ejection fraction; GFR, glomerular filtration rate; HR, heart rate; HF, heart failure; ICU, intensive care unit; IVP, intravenous vasopressor; LOS, length of stay; MAOI, monoamine oxidase inhibitors; MAP, mean arterial pressure; mcg, microgram; MICU, medical intensive care unit; MID, midodrine; min, minute; mmol, millimole; NE, norepinephrine; NR, not reported; PAD, peripheral arterial disease; PTA, prior to admission; RCT, randomized-controlled trial; SBP, systolic blood pressure; SICU, surgical intensive care unit; SCr, serum creatinine; TCA, tricyclic antidepressants; TICU, trauma intensive care unit; USA, United States of America.

### Patient Characteristics

The most common admitting unit was a medical or mixed ICU (11/15; 73%) followed by a trauma/surgical ICU (7/15; 47%); many included both ICU types (Table 2). The most common shock type was “mixed” which included cardiogenic, spinal, septic, and postoperative shock/hypotension cases into one category (7/15; 47%) followed by septic only (5/15; 30%). One (7%) study did not report shock type. Severity of illness was defined using APACHE II, III or IV in ten (67%) studies, Euroscore in one (7%), and SOFA score in one (7%); severity of illness was not reported in three (20%) studies. Patients with renal insufficiency, ranging from chronic kidney disease to acute kidney injury, were excluded from seven (47%) studies.

**Table 2.** Patient characteristics and outcomes of included studies

**Study**	**Subjects**	Illness Severity^*^	**Shock Type**	Renal Function^*^ (SCr in mg/dL)	**Level of Care**	**ICU LOS, d Hospital LOS, d**	**ICU Mortality, n (%)** **Hospital Mortality, n (%)**
Ahmed Ali 2022	TICU n=30 MID n=30 Control	NR	Spinal	MID first day SCr 0.72 ± 0.39 Control first day SCr 1.02 ± 0.59 p=0.005 MID last day SCr 1.04 ± 0.62 Control last day SCr 1.39 ± 1.27 p=0.276	ICU	ICU MID 5.13 ± 1.83 Control 9.03 ± 3.74 p<0.001 Hospital—NR	NR
Costa-Pinto 2022	MICU n=32 MID n=30 Control	APACHE III MID 49.5 (41, 56.25) Control 48.5 (38.25, 58) p=0.76	Septic; post-op	MID SCr 0.82 (0.66, 1.17) Control SCr 0.83 (0.64, 1.00) p=0.53	ICU	ICU MID 2.08 (1.06, 3.08) Control 2.46 (1.6, 3.89) p=0.14 Hospital MID 9 (5.75, 25.25) Control 7.5 (6, 14.5) p=0.92	ICU MID 1 (3.1%) Control 0 (0%) p>0.99 Hospital MID 3 (9.4%) Control 2 (6.7%) p>0.99
Davoudi-Monfared 2021	General ICU n=15 MID n=13 Control	APACHE II MID 17.06 ± 3.15 Control 16.15 ± 4.01 p=0.10 SOFA MID 7.5 ± 2.17 Control 8.3 ± 2.25 p=0.99	Septic	MID SCr 1.2 (0.9,1.7) Control SCr 1.3 (0.85, 1.95) p=0.95	ICU	ICU MID 8 (4, 15) Control 12 (4.5, 20) p=0.55 Hospital—NR	ICU—NR Hospital (28-d) MID 8 (55.4%) Control 9 (69.2%) p=0.32
Hussein El Adly 2022	General ICU n=30 MID n=30 Control	APACHE II^**^ MID 24 (13–39) Control 21.5 (7–39) SOFA^**^ MID 11.5 (13–39) Control 9 (3–20)	Septic	NR	ICU	ICU Control 11.9 ± 7 MID 11.5 ± 6.8 p=0.876 Hospital—NR	ICU Control 22 (73.3%) MID 13 (43.4%) p=0.018 Hospital—NR
Kim 2021	ICU to Floor n=19 MID n=132 Control	NR	NR	NR	Floor (post-ICU)	ICU MID 4.1 ± 3.8 Hospital MID 13.3 ± 12.2	ICU—NR Hospital Association between MID and mortality: OR 7.5 (1.3–44.5); p=0.03
Lal 2021	MICU n=17 MID n=15 Placebo	SOFA MID 6.8 ± 3.3 Placebo 6.3 ± 2.6 p=0.64	Septic	MID SCr 2.0 ± 0.9 Placebo SCr 1.4 ± 0.6 p=0.03	ICU	ICU MID 2.29 (1.5, 3.9) Placebo 2.45 (1.6, 3.2) p=0.36 Hospital MID 7 (3.5, 10.5) Placebo 7 (4, 12) p=0.41	NR
Levine 2013	SICU n=20 MID	APACHE II MID 18 ± 6	Post-op	MID SCr 0.74 ± 0.28	ICU	ICU time from MID initiation to discharge 4 (3, 6) Hospital time from MID initiation to discharge 8.5 (5, 16)	ICU 1 (5%) Hospital 1 (5%)
Macielak 2021	General ICU n=33 MID Floor n=11 MID	NR	NR	MID SCr 1.56 (0.85, 2.33)	Any	ICU 12 (5, 27) Hospital—NR	ICU—NR Hospital 13 (29.5%)
Poveromo 2016	MICU, SICU, CVICU, NICU, TICU n=94 MID n= 94 Control	APACHE IV MID 59 (44, 83) Control 82 (66, 93) p=0.02	Cardiogenic; Spinal; Post-op; Septic	NR	ICU	ICU MID 5.5 (3, 14.8) Control 5 (3, 10) p=0.29 Hospital MID 12 (8, 21.8) Control 9.5 (5, 16) p<0.01	ICU—NR Hospital MID 8 (8.5%) Control 21 (22.3%) P=0.01
Rizvi 2018	MICU, SICU, CTICU, TICU, NICU, CICU n=1119 MID n=456 no IVP n=663 yes IVP	APACHE III MID (no IVP) 76 (62, 93) MID (yes IVP) 78 (62, 96)	Cardiogenic; Spinal; Septic	SCr before MID: 1.96 SCr 24 h after MID: 1.94 p=0.3	ICU	ICU MID (no IVP) 4 (2, 9) MID (yes IVP) 6 (3, 14) Hospital MID (no IVP) 15 (8, 31) MID (yes IVP) 18 (8, 37)	ICU MID (no IVP) 35 (8%) MID (yes IVP) 74 (11%) Hospital MID (no IVP) 77 (17%) MID (yes IVP) 129 (19%)
Rizvi 2019	MICU, SICU, CTICU, TICU, NICU, CICU n=1010 MID	APACHE III MID 78 ± 25.6	Cardiogenic; Septic	NR	ICU	ICU MID continued at ICU discharge 8.5d ± 10.7 MID stopped at ICU discharge 10.6 ± 13.4 Hospital—NR	ICU—NR Hospital MID continued at ICU discharge HR 0.45 (0.30–0.68), p<0.001 1-year MID continued at ICU discharge HR 1.56 (1.23–1.99) p<0.001
Santer 2020	SICU, MICU n=66 MID n=66 Placebo	APACHE II MID 14.7 ± 5.5 Placebo 14.8 ± 5.9	Septic; Post-op; Other	MID SCr 0.8 (0.6, 1.0) Placebo SCr 0.9 (0.6, 1.3)	ICU	ICU MID 6 (5, 8) Placebo 6 (4, 8) p=0.46 Hospital MID 11 (9, 21) Placebo 14 (9, 22) p=0.45	NR
Tremblay 2020	CTICU n=74 MID n=74 Control	Euroscore II MID 1.94 (1, 2.91) Control 2.08 (1.31, 4) p=0.088	Vasoplegia after cardiac surgery	Acute kidney injury: MID 11 (14.9%) Control 10 (13.5%) p=0.462	ICU	ICU MID 4.13 (2.83, 6.08) Control 2.83 (2, 4.13) p=0.001 Hospital—NR	ICU—NR Hospital MID 10 (13.5%) Control 1 (1.4%) p=0.036
Whitson 2016	MICU n=135 MID n=140 Control	APACHE IV MID 82.6 ± 26.4 Control 84.3 ± 26.8 p=0.55	Septic	Change in SCr: MID 0.5 ± 1.3 Control 0.8 ± 1.6 p=0.048	ICU	ICU MID 7.5 ± 5.9 Control 9.4 ± 6.7 p=0.017 Hospital MID 21.9 ± 14.4 Control 24.2 ± 14.3 p=0.3	ICU MID 15 (11.1%) Control 26 (18.6%) p=0.08 Hospital MID 31 (23%) Control 32 (25.7%) p=0.6
Wood 2022	SICU, MICU n=19 MID n=42 Control	APACHE II MID 15 (12, 17) Control 18.5 (17, 25)	Septic, Post-op, Other	NR	ICU or step down unit	ICU MID 7 (6, 13) Control 6 (5, 6) p=0.0058 Hospital MID 26 (14, 51) Control 14 (10, 17) p=0.022	NR

Medians reported as value (IQR); means reported as value ± SD;

* baseline values unless otherwise specified;

** reported as range instead of IQR.

Abbreviations: APACHE, Acute Physiology and Chronic Health Evaluation; CTICU, cardiothoracic surgery intensive care unit; d, day(s); h, hour(s); ICU, intensive care unit; IVP, intravenous vasopressor; LOS, length of stay; MICU, medical intensive care unit; MID, midodrine; NICU, neurological intensive care unit; NR, not reported; post-op, post-operative; PTA, prior to admission; SCr, serum creatinine; SICU, surgical intensive care unit; SOFA, sequential organ failure assessment; TICU, trauma intensive care unit.

### Midodrine Administration

A starting dose of 10 mg every 8 hours (4/15; 27%) or three times daily (3/15; 20%) was most common, with the exception of two (13%) studies that reported a starting dose of 20 mg every 8 hours, and six (40%) that did not report an initial dose (Table 3). A protocol for midodrine dosing was present in six (40%) studies. Seven (47%) studies used fixed dosing of 10 mg every 8 hours or three times daily and two (13%) used a fixed dose of 20 mg every 8 hours. Doses ranged from 2.5 mg every 12 hours (5 mg total daily dose) to 20 mg every 6 hours to 40 mg every 8 hours (120 mg total daily dose).

**Table 3.** Midodrine Use

**Study**	**Protocol**	**Protocol details**	**Initial Dose/Frequency**	**Max Dose/Frequency**	**Titration vs. Fixed Dose**	**Start Before, With or After Pressors**	**Duration of Midodrine (d)**	**Route of Admin**	**Continued at ICU Discharge n (%)**	**Continued at Hospital Discharge n (%)**
Ahmed Ali 2022	Yes	4 doses of MID, then IVP weaning initiated	10 mg every 8 h	10 mg every 8 h	Fixed	After	NR	PO	No	No
Costa-Pinto 2022	Yes	MID administered until off IVP for at least 24 h Wean: 7.5 mg every 8 h for 24 h, then 5 mg every 8 h for 24 h, then DC	10 mg every 8 h	10 mg every 8 h	Fixed	After	NR	NR	Yes	NR
Davoudi-Monfared 2021	Yes	Randomly assigned to adjunctive MID to facilitate IVP wean	10 mg TID	10 mg TID	Fixed	With	Up to 5 d	If conscious, PO; if not, via NGT	NR	NR
Hussein El Adly 2022	Yes	Randomly assigned to adjunctive midodrine to facilitate IVP wean	10 mg TID	10 mg TID	Fixed	After	NR	PO tablet or crushed (via Ryle)	NR	NR
Kim 2021	No	No protocol	NR	NR	NR	NR	NR	NR	19 (12.6)	NR
Lal 2021	Yes	If septic shock without response to antibiotics and fluids, randomized to MID or placebo	10 mg every 8 h	10 mg every 8 h	Fixed	After or monotherapy	3 doses	PO	No	No
Levine 2013	No	No protocol	NR	20 mg TID	Titration; no details	After	4 (3–7)	PO	NR	NR
Macielak 2021	No	No protocol	NR	20 mg every 6 h	Titration	After n=23 (52.3%) Continued from home n=18 (40.9%) Monotherapy n=3 (6.8%)	NR	NR	Yes	Yes
Poveromo 2016	No	No protocol	NR	10 mg every 4 h	Titration	After	4.4 (3.2, 7.8)	NR	NR	NR
Rizvi 2018	No	No protocol	NR	30 mg every 8 h	Titration	After (59%); Before (41%)	NR	PO	NR	NR
Rizvi 2019	No	No protocol	NR	40 mg every 8 h	Titration	After: 58% Before or monotherapy: 42%	11.8 ± 20.9	PO	672 (67)	311 (34)
Santer 2020	Yes	Randomized to MID or placebo until ICU discharge. DC’ed with stable at goal blood pressure at discretion of clinical team per a standardized weaning protocol (decrease dose every 1–2 d from 20 mg to 10 mg every 8 h, then 5 mg every 8 h, then DC)	20 mg every 8 h	20 mg every 8 h	Fixed	After at least 24 h of IVP	1.77 (0.98, 2.97)	PO	NR	No
Tremblay 2020	No	No protocol	10 mg TID (for n=61, 82.4%)	Only n=2 with doses >10 mg; All TID	Majority fixed. Progressive tapering for n=19 (26%)	After at least 12 h of IVP	1.67 (0.96, 3.04)	NR	17 (23)	NR
Whitson 2016	No	No protocol	10 mg every 8 h	40 mg every 8 h	Titration	After at least 24 h of IVP	6.15 For patients who were not discharged on MID (n=117, 86.7%)	NR	Yes	18 (13.3)
Wood 2022	No	Started on MID at discretion of treatment team. If enrolled, MID administered until at least 24 h after DC of IVP	20 mg every 8 h	20 mg every 8 h	Fixed	After	NR	PO	NR	NR

Medians reported as value (IQR); means reported as value ± SD. Abbreviations: d, day(s); DC, discontinued or discontinuation; h, hour(s); ICU, intensive care unit; IVP, intravenous vasopressors; MID, midodrine; mg, milligrams; NGT, nasogastric tube; NR, not reported; PO, by mouth; TID, three times daily

No study adjusted the midodrine dose for renal function though one did recommend a lower starting dose for patients with kidney dysfunction [27]. Most studies reported administering midodrine orally (7/15; 47%), but many did not specify if this was given by mouth or through a feeding tube, and only two explicitly stated they crushed or administered it via gastrostomy tube [18,19]. Midodrine was initiated in the ICU in a majority of publications (14/15; 93%). No studies included patients in the emergency department.

Two (13%) studies specified weaning protocols for midodrine including decreasing the dose from 10 mg to 7.5 mg every 8 hours for 24 hours, then 5 mg every 8 hours for 24 hours, then discontinuation or decreasing the dose every 1–2 days from 20 mg to 10 mg every 8 hours, then 5 mg every 8 hours, then discontinuation [16,23]. Six studies (40%) reported midodrine continuation past ICU discharge (range: 13–67% of patients) and three (20%) studies reported it was continued at hospital discharge (range: 13–52% of patients).

### Intravenous Vasopressors

Midodrine was initiated to wean off IVPs during shock resolution in most studies (11/15; 73%) with a minority describing its use before or with IVPs during the early phase of shock (5/15; 30%). Nine (60%) studies reported that all patients were on IVPs when midodrine was initiated, and in the other six studies, 48% to 59% of patients were being treated with IVPs (Table 4). The number of IVPs administered at midodrine initiation was reported in ten (67%) studies and most (8/10; 80%) reported only one IVP (norepinephrine, phenylephrine, or metaraminol). The median dose of IVPs, expressed as norepinephrine equivalents, was 0.08 (0.05–0.14) mcg/kg/min. No study exclusively looked at patients not on IVPs and only two (13%) specified a weaning procedure for IVPs [15,18].

**Table 4.** Intravenous Vasopressor Use

**Study**	**Percent of patients on IVP at MID initiation, n (%)**	**Number of IVP at MID initiation**	**NEE at MID initiation**	**Time to IVP discontinuation (h)**	**Need to restart IVP, n (%)**
Ahmed Ali 2022	MID 30 (100) Control 30 (100)	1 (NE only)	NR; inclusion criteria <8 mcg/min NE	MID 79.2 ± 31.7 Control 166.3 ± 55.7 p<0.001	NR
Costa-Pinto 2022	MID 32 (100) Control 30 (100)	1 (NE or metaraminol)	NR; inclusion criteria <10 mcg/min NE or <100 mcg/min metaraminol	MID 16.5 (7.2, 27.5) Control 19 (12.2, 38.5) p=0.32	MID 6 (18.8) Control 4 (13.3) p=0.73
Davoudi-Monfared 2021	MID 15 (100) Control 13 (100)	1 (NE only)	Midodrine median NEE 0.14 mcg/kg/min Control median NEE 0.13 mcg/kg/min	MID 96 (48, 192) Control 120 (72, 264) p=0.36	MID 4 (26.7) Control 5 (38.5) p=0.39
Hussein El Adly 2022	MID 30 (100) Control 30 (100)	1 (NE only)	Midodrine median NEE 0.08, range 0.04–0.21 mcg/kg/min Control median NEE 0.11, range 0.02–0.35 mcg/kg/min	MID 26 (14, 106) Control 78.5 (32, 280) p<0.001	MID 3 (10%) Control 3 (10%)
Kim 2021	All 73 (48.3)	NR	NR	NR	NR
Lal 2021	MID 11 (52.4) Placebo 10 (47.6)	NR	NR	Requiring IVP at 12 h: MID 41.2% vs Control 60% p=0.29	NR
Levine 2013	MID 20 (100)	1 (NE or PE)	Midodrine mean NEE 4.1 mcg/min	MID 17 (7, 38.4)	NR
Macielak 2021	MID 23 (52.3)	NR	Midodrine mean NEE 0.1 mcg/kg/min	NR	NR
Poveromo 2016	MID 94 (100) Control 94 (100)	MID: 1 (40.4%), 2 (41.5%), 3+ (18.1%) Control: 1 (62.8%), 2 (24.4%), 3+ (12.8%)	Midodrine median NEE 0.05 (0.03, 0.08) mcg/kg/min Control median NEE 0.05 (0.03, 0.08) mcg/kg/min	MID 28.8 (12, 67.2) Control: NR	MID 42 (44.7) Control: NR
Rizvi 2018	MID 663 (59.0)	NR	Midodrine median NEE 0.24 mcg/kg/min	Requiring IVP at 24 h: 48%	NR
Rizvi 2019	MID 587 (58.1)	NR	Midodrine median NEE 0.19 mcg/kg/min	NR	NR
Santer 2020	MID 66 (100) Placebo 66 (100)	1 (NE, PE, or metaraminol)	Midodrine median NEE 0.03 (0.02, 0.06) mcg/kg/min Control median NEE 0.03 (0.02, 0.06) mcg/kg/min	MID 23.5 (10.4, 44) Control 22.5 (10.4, 40) p=0.62	NR
Tremblay 2020	MID 74 (100) Control 74 (100)	MID: 1 (85.1%), 2 (13.5%), 3 (1.4%) Control: NR	All patients median NEE 0.05 (0.03, 0.09) mcg/kg/min	MID 19 (4, 44)	MID 16 (21.6)
Whitson 2016	MID 135 (100) Control 140 (100)	1 (NE or PE)	Midodrine mean NEE 0.09 mcg/kg/min Control mean NEE NR	MID 69.6 ± NR Control 91.2 ± NR p<0.001	MID 7 (5.2) Control 21 (15) p=0.007
Wood 2022	MID 19 (100) Control 42 (100)	1 (NE or metaraminol)	Midodrine median NEE 0.05 mcg/kg/min Control median NEE 0.08 mcg/kg/min	MID 26 (22, 36) Control 24 (17, 93) p=0.511	NR

Medians reported as value (IQR); means reported as value ± SD. Abbreviations: IVP, intravenous vasopressors; kg, kilogram; mcg, microgram; MID, midodrine; min, minute; NE, norepinephrine; NEE, norepinephrine equivalents; NR, not reported; PE, phenylephrine.

There were no reports of IVP-related or central venous catheter-related complications. Only one (6%) study reported that the midodrine group required a shorter duration of central venous catheterization, but the finding was not statistically significant [21]. The time to IVP discontinuation, the most common primary outcome studied, was 26 (20.1–59.4) hours for the midodrine patients and 78.5 (23.3–105.6) for controls.

### Adverse Drug Effects

Thirteen (87%) studies reported the incidence of bradycardia with six (46%) reporting it was present (Table 5). The definition for bradycardia varied and was generally defined as a heart rate <40–60 beats per minute. Of the 204 individual patients with bradycardia, only one (0.5%) required midodrine discontinuation and none required a medical intervention (e.g., atropine) [4].

**Table 5.** Reported Side Effects

**Study**	**Bradycardia Definition**	**Bradycardia Incidence, n (%)**	**Heart Rate Change (bpm)**	**Bradycardia Interventions**	**Bowel Ischemia n (%)**	**Peripheral Ischemia n (%)**	**Cerebral Ischemia n (%)**	**Allergy n (%)**
Ahmed Ali 2022	No definition	NA	MID Day 1: 117 ± 14.2, MID Mid-study: 103.77 ± 16.65, MID Last Day: 79 ± 16.9 Control Day 1: 120.43 ± 14.64 Control Mid-study: 97.1 ± 16.65 Control Last Day: 96.73 ± 18.75	NA	NR	NR	NR	NR
Costa-Pinto 2022	Bradycardia: ≤50 bpm; Severe bradycardia: <40 bpm	Bradycardia within 24 h: MID 10 (31.2) Control 2 (6.7) p=0.02	Baseline MID HR: 76 (70, 85) Baseline Control HR: 77.5 (65.5, 85) p=0.61 MID HR over 24 h: 69 (62, 82) Control HR over 24 h: 74 (67, 83) p=0.21	None; episodes of bradycardia, except one, were transient and deemed clinically insignificant	NR	NR	NR	NO
Davoudi-Monfared 2021	<60 bpm	NO	NR	NA	NR	NR	NR	NR
Hussein El Adly 2022	<50 bpm	NR	NR	NA	NR	NR	NR	NR
Kim 2021	NR	NR	NR	NA	NR	NR	NR	NR
Lal 2021	<40 bpm and symptomatic	NO	NR	NA	NO	NO	NO	NO
Levine 2013	No definition	NR	Before MID HR 82 ± 13 After MID HR 81 ± 15 p=0.66	NA	NR	NR	NR	NR
Macielak 2021	<50 bpm	NO	NR	NA	1 (2.3)	NO	NR	NR
Poveromo 2016	<60 bpm for two consecutive readings	MID: 12 (12.8) Control: NR	NR	NR	NR	NR	NR	NR
Rizvi 2018	≤50 bpm; ≤40 bpm	≤50 bpm: 172 (15.4) ≤40 bpm: 100 (9) Lowest HR: 39 (33, 44) bpm	NR	None	2 (0.18)	NR	NO	NR
Rizvi 2019	NR	NR	NR	NA	NR	NR	NR	NR
Santer 2020	<40 bpm or ≥20% decrease from a pre-specified goal	MID: 5 (7.6) Control: 0 (0) p=0.02	NR	NR	NR	NR	NR	NR
Tremblay 2020	No definition	NR	NR	NA	2 (2.7)	NR	NR	NR
Whitson 2016	No definition	MID: 1 (0.7) Control: NO	NR	MID discontinued and bradycardia resolved.	NR	NR	NR	NR
Wood 2022	<40 bpm or ≥20% decrease from a pre-specified goal	MID: 4 (22) Control: 1 (2.4) p=0.025	No significant change	NR	NR	NR	NR	NR

Medians reported as value (IQR); means reported as value ± SD; Abbreviations: bpm, beats per minute; MID, midodrine; HR, heart rate; NR, not reported; NA, not applicable; NO, not observed; SCr, serum creatinine.

Three (20%) studies reported the incidence of hypertension using various definitions, most commonly a systolic blood pressure >160 mmHg. The incidence of hypertension ranged from 5.6%–10.6% in the studies that reported it. None of the studies reported hypertension as a reason for midodrine discontinuation.

Four (27%) studies assessed for ischemia, either mesenteric or peripheral, with limited description on how it was assessed. Five (5/1128; 0.4%) patients in the four studies developed mesenteric ischemic requiring midodrine discontinuation. Three of the five had alternative explanations (e.g., multiple high-dose IVPs) but two did not. No peripheral (e.g., digits and limb) ischemia was observed.

### Cost Analyses

One study conducted a cost analysis and reported direct medical cost per day in midodrine patients was $2,776.50 compared to $2,454.00 in control patients. Indirect medical costs were not considered.^18^

### Quality of Evidence Assessment

Evaluation using the JBI Critical Appraisal Checklist criteria for randomized controlled trials, case control studies, case series, and cohort studies, revealed varied adherence to bias-reducing strategies within individual study designs (Table 6). Few studies (5/15, 33.3%) met all bias-reduction criteria for their study type, with the majority of studies (10/15, 66.7%) being at risk for the introduction of bias in at least one facet of the study [2,22,23,24,26]. Importantly, five of six randomized controlled studies were at significant risk of bias with only one study employing all assessed methods of bias reduction [15,16,18,19,21,23].Full details of quality assessments are provided in Table 6.

**Table 6.** Quality appraisal for included studies by study design

**Randomized controlled trials**												
**Study**	**Randomization**	**Allocation concealment**	**Groups similar at baseline**	**Participants blinded**	**Staff delivering treatment blinded**	**Groups treated the same except intervention**	**Blinded outcomes assessors**	**Standardized outcomes measurement**	**Complete follow-up or differences described, analyzed**	**Participants analyzed in randomization group**	**Appropriate statistics**	**Design appropriate and deviations from standard accounted for**
Ahmed Ali 2022	Yes	No	No	Unclear	No	Yes	Unclear	Yes	Yes	Yes	Yes	Yes
Costa-Pinto 2022	Yes	Yes	Yes	Unclear	No	Yes	Unclear	Yes	Yes	Yes	Yes	Yes
Davoudi-Monfared 2021	Yes	Unclear	Yes	Unclear	Unclear	Yes	Unclear	Yes	Yes	Yes	Yes	Yes
Hussein El Adly 2022	Yes	Yes	Yes	No	No	Yes	Unclear	Yes	Yes	Yes	Yes	Yes
Lal 2021	Yes	Yes	No	Yes	Yes	Yes	Yes	Yes	Yes	Yes	Yes	Yes
Santer 2020	Yes	Yes	Yes	Yes	Yes	Yes	Yes	Yes	Yes	Yes	Yes	Yes

**Cohort Studies**											
**Study**	**Groups similar, from same population**	**Exposures measured similarly**	**Exposure measurement reliable, valid**	**Confounders identified**	**Strategies to address confounders described**	**Groups free of outcome at start**	**Outcomes measurement reliable, valid**	**Follow-up time reported, sufficient for outcome to develop**	**Follow-up complete or loss reasons described**	**Strategies to address incomplete followup used**	**Appropriate statistics**
Kim 2021	NA	NA	Yes	No	No	Yes	Yes	Yes	Yes	NA	Yes
Levine 2013	NA	NA	Yes	Yes	Yes	Yes	Yes	Yes	Yes	NA	Yes
Macielak 2021	NA	NA	Yes	Yes	Yes	Yes	Yes	Yes	Yes	NA	Yes
Poveromo 2016	Yes	Yes	Yes	Yes	No	Yes	Yes	Yes	Yes	NA	Yes
Tremblay 2020	Yes	Yes	Yes	Yes	Yes	Yes	Yes	Yes	Yes	NA	Yes
Whitson 2016	Yes	Yes	Yes	No	No	Unclear	Yes	Yes	Yes	NA	Yes

**Case Control Studies**										
**Study**	**Comparable groups**	**Cases, controls appropriately matched**	**Consistent criteria to ID cases, controls**	**Exposure measurement standard, valid, reliable**	**Exposure measurement standard for cases, controls**	**Confounders identified**	**Strategies to address confounders described**	**Outcomes measurement standard, reliable, valid for cases, controls**	**Exposure period long enough**	**Appropriate statistics**
Wood, 2022	No	Unclear	No	Yes	Yes	Yes	Yes	Yes	Yes	Yes

**Case Series**										
**Study**	**Clear inclusion criteria**	**Condition measurement reliable, valid**	**Valid methods of case identification**	**Consecutive case inclusion**	**Complete inclusion of participants**	**Clear demographic reporting**	**Clear clinical info reporting**	**Outcomes or follow-up results clearly reported**	**Clear reporting of site demographics**	**Appropriate statistics**
Rizvi, 2018	Yes	Yes	Yes	Yes	Yes	Yes	Yes	Yes	Yes	Yes
Rizvi, 2019	Yes	Yes	Yes	Unclear	Yes	Yes	Yes	Yes	Yes	Yes

Abbreviation: NA, not applicable.

## Discussion

This systematic review included 15 publications and 1,714 patients who received midodrine for shock and is the first to focus on the practical aspects of midodrine use. Important findings included the predominance of observational studies (60%) conducted at single centers (80%), reliance on a fixed dose of 10 mg three times daily or every 8 hours (47%), absence of a midodrine dosing protocol and wide variability in dose administered (5 to 120 mg total daily dose). Additionally, no study adjusted the dose for renal dysfunction, looked exclusively at patients not on IVPs, described IVP-related or central venous catheter-related complications, or included patients in an emergency department or rural setting. These findings suggest caution be used when interpreting or applying the existing data regarding midodrine use for shock.

Midodrine was used for a variety of shock types, including cardiogenic, spinal, septic, and post-operative shock, often combining all shock patients together. The most common indication for midodrine was to decrease the duration or intensity of IVPs. Avoiding IVPs entirely would obviate the need for a central line or ICU admission, which has only been commented on by Rivzi and colleagues [2,3]. Other potential benefits of midodrine use prior to or early with IVPs include decreasing fluid requirements or IVP requirements, possibly reducing risk for adverse events from IVPs. These endpoints have been understudied and only one publication reported time to first midodrine dose (13 hours) [21]. The majority focused on late use of midodrine when patients were weaning off low doses of IVPs.

Most publications reported midodrine dosing intervals of either every 8 hours or three times a day (possibly with a 12-hour gap without doses overnight) though a recent paper reported dosing every 6 hours [22]. When midodrine was FDA approved for symptomatic orthostatic hypotension, the prescribing information recommended dosing three times a day during daylight hours due to the risk of nocturnal supine hypertension. Dosing it every 8 hours has its drawbacks as the active metabolite of midodrine, desglymidodrine, has a duration of action of 2–3 hours and a half-life of 3–4 hours, which could lead to suboptimal dosing. Midodrine may be better suited for every 4- or 6-hour dosing to maintain adequate serum concentrations as suggested in studies of orthostatic hypotension [5] but this must be studied in patients with shock.

Most publications reported using midodrine in fixed doses. Intravenous vasopressors are titrated to an objective endpoint (e.g., mean arterial pressure); logically midodrine should be titrated to effect as well, supported by its FDA approved dosing. The studies that utilized dose titrations did not have protocols or guidance for how midodrine was titrated. Similarly, none of the randomized controlled trials allowed dose titrations, which raises the question of whether their overall negative findings would be different with titratable, optimized dosing protocols [6,7].

None of the included studies adjusted midodrine dosing for hepatic or renal dysfunction and those with end-organ injury were often excluded. The FDA label lists acute renal disease as a contraindication for use. Despite this, midodrine is commonly used to treat vasodilatory shock in patients with cirrhosis or during renal replacement therapy [28,29,30]. The lack of information related to the pharmacokinetics of midodrine absorption in shock or accumulation with repeated dosing in patients with renal or hepatic dysfunction should be addressed in future trials. The bioavailability of midodrine is 93% and not affected by food in healthy volunteers but no study has been conducted during critical illness or compared oral versus gastric tube administration.

Previously published meta-analyses reported findings focused on safety and effectiveness [8,9,10]. Our systematic review builds upon their findings by answering questions about the bedside approach to midodrine use and the clinical relevance of its adverse drug effects. We determined that starting doses of 10–20 mg every 6 to 8 hours are most commonly prescribed. Although the studies included both fixed and titrated dosing, titration to an objective endpoint is prudent given the pharmacodynamic and pharmacokinetic properties of midodrine and the proven dose-response for blood pressure. Utilization of dosing protocols for IVPs and midodrine might improve the safety and effectiveness of both.

This systematic review has limitations, one of which is its semi-quantitative design. We chose not to pursue a full meta-analysis due to the heterogeneity and low quality of the data available. Though RCTs are generally considered the highest quality of evidence, many midodrine studies were unblinded, used fixed doses or dosing intervals twice as long as shown to be effective [2,3,4,5,15,16,17,18,19,20,21,22,23,24,25,26]. Accordingly, we felt that including a broader sample of published data may provide additional information despite potential decreases in quality. We acknowledge that norepinephrine equivalents may have variability based on differences in base formulation [31]. Our aggregation and interpretation of adverse drug effects was limited by the specificity with which they were reported; under reporting is likely with retrospective reviews. Additionally, definitions of how adverse effects were identified or defined varied and were sometimes absent altogether.

There are many possible directions for future investigators of midodrine's utility for shock including evaluating fixed versus titrated dosing, optimal dosing frequency (every 4, 6 or 8 hours), early initiation in the emergency department, use in rural hospital settings, pharmacokinetic studies of oral versus gastric tube administration, and endpoints related to avoiding IVPs, central venous catheters, and their related complications. In summation of the studies reviewed, we propose specific clinical scenarios and conditions where midodrine may either be considered for use or alternatively should be avoided (Table 7). However, overall, a better understanding of the optimal dosing strategy, pharmacokinetics, and clinical effectiveness of midodrine in the setting of shock is needed and should be a priority for investigators.

**Table 7.** Where midodrine may be consider and avoided

**Some Experience – Likely Safe**	**Limited Experience – Use Caution**	**No Experience – Avoid Use**	**Contraindications for Use**
Orthostatic hypotension	Vasopressor sparing	Cardiogenic shock	Pheochromocytoma
Hemodialysis hypotension	Mixed shock	Cerebral vasospasm	Thyrotoxicosis
Septic Shock	Renal failure	Unknown enteral absorption	Urinary retention
Vasopressor weaning	Lactate clearance	Mechanical circulatory support	
Hepatorenal syndrome	Bradycardia	Daily dose >120 mg	
Fixed dosing regimen	Dosing every four hours		
	Hepatic impairment		
	Titrated dosing regimen		

## Conclusion

The literature describing midodrine for blood pressure augmentation in shock is heterogeneous and comprised of mostly low-quality data, creating opportunities for future investigations. Controlled trials should carefully account for midodrine's initiation thresholds, dose titration strategies, and the clinical relevance of adverse drug effects to better describe its safety and effectiveness in shock.
